# Cell loaded hydrogel containing Ag‐doped bioactive glass–ceramic nanoparticles as skin substitute: Antibacterial properties, immune response, and scarless cutaneous wound regeneration

**DOI:** 10.1002/btm2.10386

**Published:** 2022-08-03

**Authors:** Esmaeel Sharifi, Seyede Athar Sadati, Satar Yousefiasl, Rossella Sartorius, Mahdi Zafari, Leila Rezakhani, Morteza Alizadeh, Ehsan Nazarzadeh Zare, Shadi Omidghaemi, Fatemeh Ghanavatinejad, Mohammad‐Saeid Jami, Erfan Salahinejad, Hadi Samadian, Ana Cláudia Paiva‐Santos, Piergiuseppe De Berardinis, Abbas Shafiee, Franklin R. Tay, Samiramis Pourmotabed, Pooyan Makvandi

**Affiliations:** ^1^ Cellular and Molecular Research Center, Basic Health Sciences Institute Shahrekord University of Medical Science Shahrekord Iran; ^2^ Department of Tissue Engineering and Biomaterials, School of Advanced Medical Sciences and Technologies Hamadan University of Medical Sciences Hamadan Iran; ^3^ School of Dentistry Hamadan University of Medical Sciences Hamadan Iran; ^4^ Institute of Biochemistry and Cell Biology (IBBC) National Research Council (CNR) Naples Italy; ^5^ National Cell Bank, Pasteur Institute of Iran Tehran Iran; ^6^ Fertility and Infertility Research Center Health Technology Institute, Kermanshah University of Medical Sciences Kermanshah Iran; ^7^ Department of Tissue Engineering, School of Medicine Shahroud University of Medical Sciences Shahroud Iran; ^8^ School of Chemistry Damghan University Damghan Iran; ^9^ Faculty of Materials Science and Engineering K. N. Toosi University of Technology Tehran Iran; ^10^ Dental Implants Research Center Hamadan University of Medical Sciences Hamadan Iran; ^11^ Department of Pharmaceutical Technology, Faculty of Pharmacy University of Coimbra Coimbra Portugal; ^12^ REQUIMTE/LAQV, Group of Pharmaceutical Technology, Faculty of Pharmacy University of Coimbra Coimbra Portugal; ^13^ UQ Diamantina Institute, Translational Research Institute, The University of Queensland Brisbane Queensland Australia; ^14^ The Graduate School, Augusta University Augusta Georgia USA; ^15^ Department of Emergency Medicine, School of Medicine Hamadan University of Medical Sciences Hamadan Iran; ^16^ Istituto Italiano di Tecnologia, Centre for Materials Interfaces Pontedera Pisa Italy

**Keywords:** Ag‐doped bioactive glass–ceramics, anti‐biofilm, hemocompatible, immunogenicity, skin substitute, wound healing

## Abstract

An ideal tissue‐engineered dermal substitute should possess angiogenesis potential to promote wound healing, antibacterial activity to relieve the bacterial burden on skin, as well as sufficient porosity for air and moisture exchange. In light of this, a glass–ceramic (GC) has been incorporated into chitosan and gelatin electrospun nanofibers (240–360 nm), which MEFs were loaded on it for healing acceleration. The GC was doped with silver to improve the antibacterial activity. The bioactive nanofibrous scaffolds demonstrated antibacterial and superior antibiofilm activities against Gram‐negative and Gram‐positive bacteria. The nanofibrous scaffolds were biocompatible, hemocompatible, and promoted cell attachment and proliferation. Nanofibrous skin substitutes with or without Ag‐doped GC nanoparticles did not induce an inflammatory response and attenuated LPS‐induced interleukin‐6 release by dendritic cells. The rate of biodegradation of the nanocomposite was similar to the rate of skin regeneration under in vivo conditions. Histopathological evaluation of full‐thickness excisional wounds in BALB/c mice treated with mouse embryonic fibroblasts‐loaded nanofibrous scaffolds showed enhanced angiogenesis, and collagen synthesis as well as regeneration of the sebaceous glands and hair follicles in vivo.

## 1 INTRODUCTION

Bacterial infection is a vicious, unaddressed problem in the healing of skin wounds. Prolonged bacterial infections are critical challenges in the management of chronic wounds. Bacterial biofilms play a significant role in persistent bacterial infections.^1^, ^2^, ^3^

Skin substitutes are acellular or cellular tissue‐engineered platforms used to restore the structure and function of the skin.^4^ They are intended for providing temporary coverage or supporting permanent wound closure. The use of skin substitutes decreases healing time, minimizes postoperative wound contraction, and enhances skin function.^5^, ^6^ An ideal skin substitute should be biocompatible, biodegradable, minimally immunogenic, mechanically stable, and keep the wound interface moist while also containing antimicrobial activities.^7^, ^8^, ^9^ Natural‐based materials (e.g., chitosan and collagen) and synthetic biocompatible polymers (e.g., polyethylene oxide) have been explored to fabricate tissue‐engineered skin scaffolds in various forms.^10^, ^11^ Fibrous and nanofibrous materials are important in bioengineering because they resemble the extracellular matrix (ECM), permeability, as well as large surface area.^12^, ^13^, ^14^ Electrospinning is a common method for fabricating nanofibrillar structures due to its simplicity and relative ease of scaling up for industrial production.^15^

Bioactive glasses and glass–ceramics (GC) are used as components of skin tissue engineering scaffolds to accelerate the healing process because of their angiogenic and anti‐inflammatory characteristics. These biomaterials promote angiogenesis via their ionic dissolution products. Bioactive glasses and GC increase the secretion of angiogenic growth factors from fibroblasts, such as vascular endothelial growth factor and basic fibroblast growth factor.^16^, ^17^, ^18^, ^19^ Trace elements such as Ca, P, Si, Cu, and Mg have also been reported to improve angiogenesis. The incorporation of Ag, Cu, or Zn improves antibacterial properties. It is possible to dope bioactive glasses and GC with these trace elements to augment these highly desirable properties.^19^, ^20^, ^21^

In light of the potential angiogenic and wound healing properties of bioactive glasses and GC, the present study deals with the fabrication of a biocompatible skin substitute with improved antibacterial properties and low immunogenicity for skin regeneration. Silver ions were incorporated into the GC composition to endow the biomaterial with bactericidal and anti‐biofilm activities. The nanofibrous scaffold was first evaluated for its physicochemical properties in vitro. An in vivo study was subsequently conducted to investigate the wound healing capability of the nanofibrous scaffold in excisional full‐thickness wounds created in BALB/c mice with or without mouse embryonic fibroblasts (MEFs).

## 2 RESULTS

### 2.1 Structural characterization of glass–ceramic powders

**FIGURE 1:**
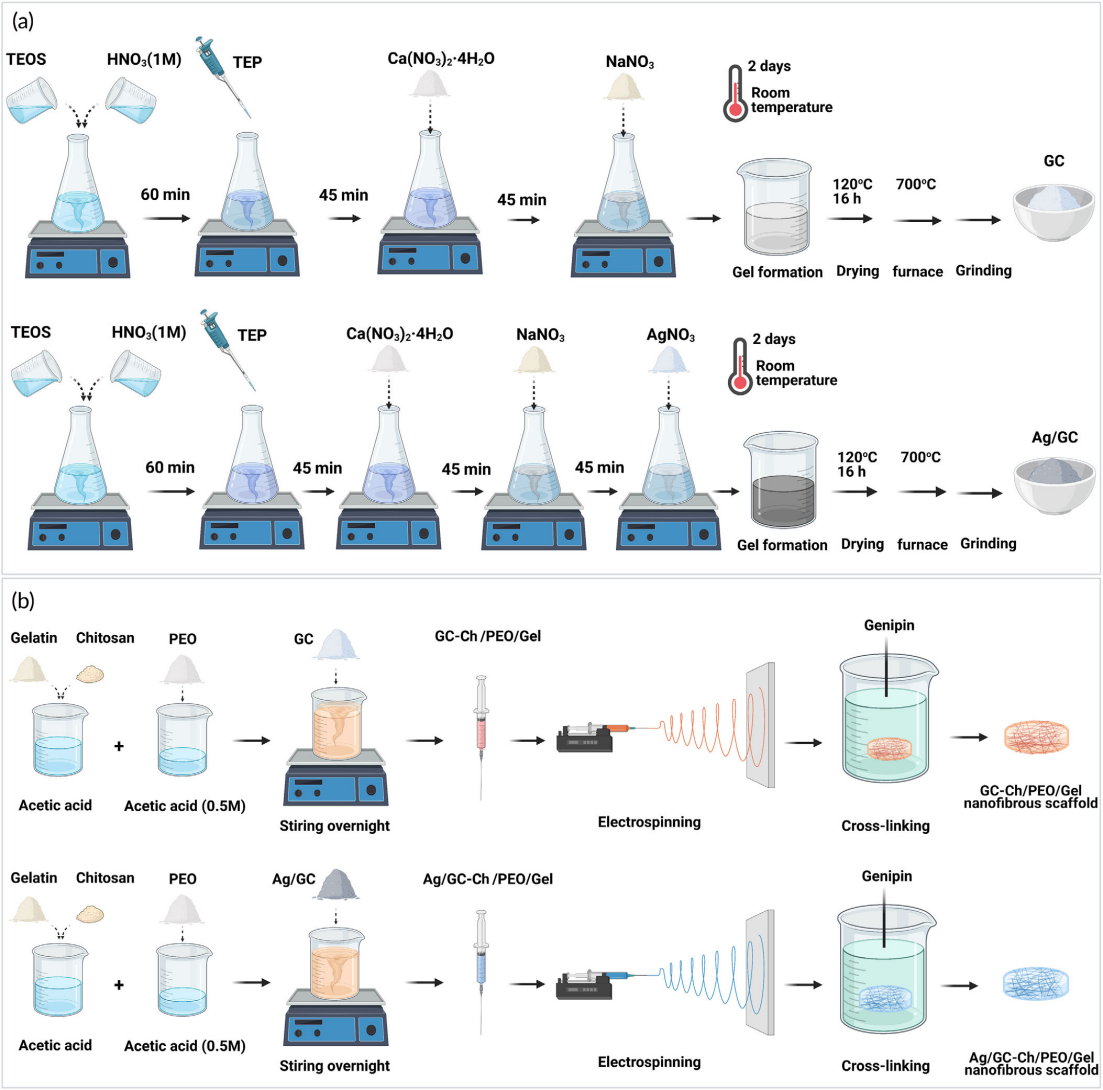
(a) GC and Ag/GC synthesis by the sol–gel method (b) GC‐Ch/PEO/Gel and Ag/GC‐Ch/PEO/Gel nanofibrous scaffold fabrication through electrospinning

**FIGURE 2:**
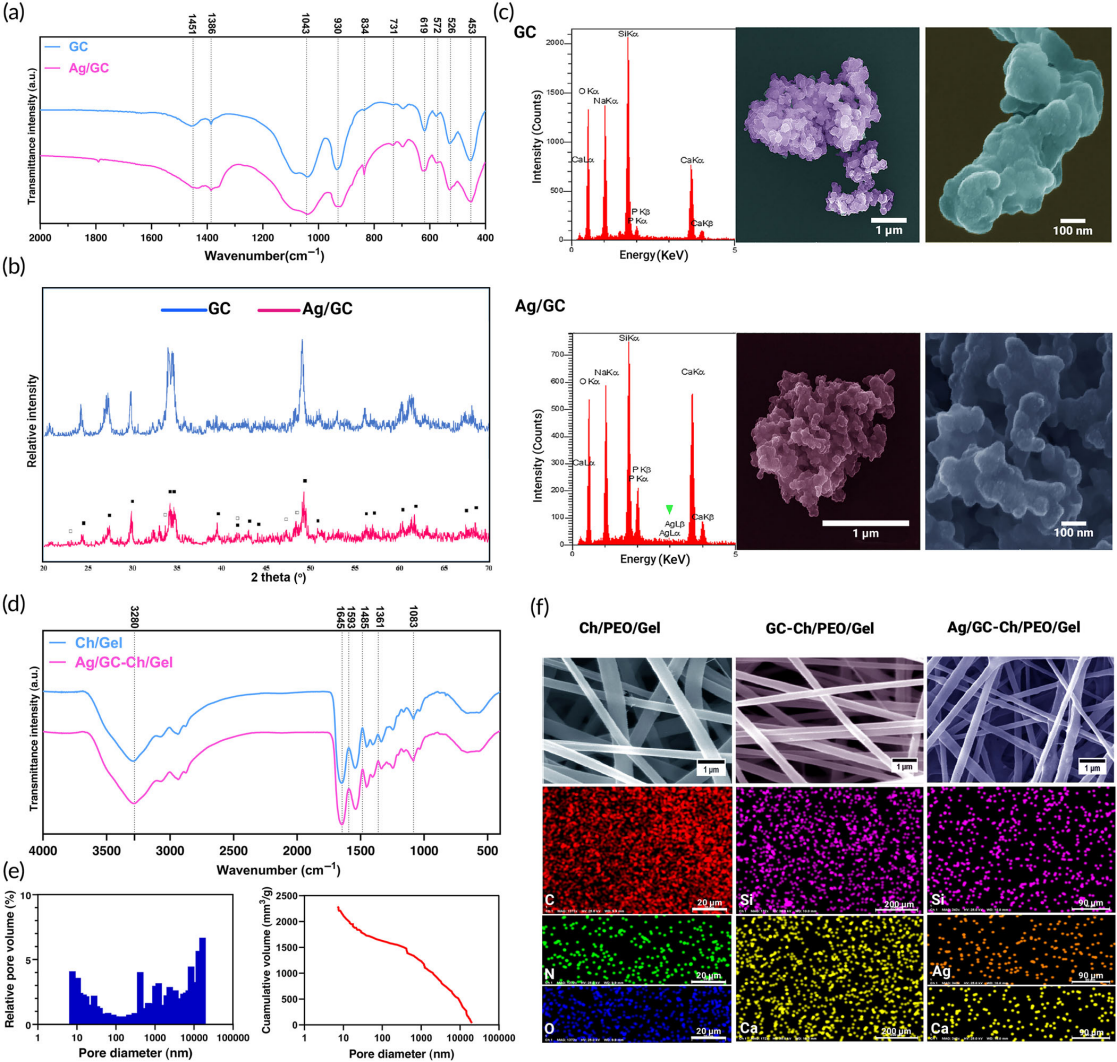
(a) Fourier transform‐infrared (FT‐IR) spectra of the prepared glass–ceramic (GC) and Ag/GC nanoparticles. (b) x‐Ray diffraction spectra of the GC and Ag/GC nanoparticles. The predominant phase, Na_6_Ca_3_Si_6_O_18_, and the secondary phase, Na_2_Ca_4_(PO_4_)_2_SiO_4_, are marked by (■) and (□), respectively. (c) Energy dispersive x‐ray spectra and field emission scanning electron microscopy (FESEM) of the sol–gel derived GC and Ag/GC nanoparticles. (d) FT‐IR spectra of the electrospun Ch/PEO/Gel and Ag/GC‐Ch/PEO/Gel scaffolds. (e) The cumulative volume of mercury in the Ag/GC‐Ch/PEO/Gel nanofibrous scaffold and the distribution of pores within the scaffolds (*n* = 3). (f) SEM micrographs and corresponding energy‐dispersive x‐ray analysis of the elemental distribution with the Ch/PEO/Gel, GC‐Ch/PEO/Gel, and Ag/GC‐Ch/PEO/Gel scaffolds

Figure 1a shows the method used for synthesizing GC and Ag/GC via sol–gel reaction. Fourier transform‐infrared (FT‐IR) spectra of the pristine and Ag‐doped GC powders are shown in Figure 2a. The peak observed at 1670 cm^−1^ was attributed to the vibrations of O—H bonds. The peak at 621 cm^−1^ was attributed to the P—O bonds. Two peaks at 926 and 1024 cm^−1^ were assigned to the Si—O tension bonds, and the peak at 460 cm^−1^ was attributed to the Si—O stretching mode. The data were suggestive of the formation of GC networks.^22^ The small peak at 644 cm^−1^ and the change in peak intensity at 930 cm^−1^ in the Ag‐doped powder was confirmative of Ag doping.

Figure 2b shows the x‐ray diffraction (XRD) pattern of the pristine GC powder and Ag‐doped GC powder. According to The International Center for Diffraction Data database, two crystalline phases were detected in each powder. The best overall matches were combeite (Na_6_Ca_3_Si_6_O_18_; Code: 01‐077‐2189, predominant phase) and silicorhenanite (Na_2_Ca_4_(PO_4_)_2_SiO_4_; Code: 00‐032‐1053). The low‐intensity noise from the XRD data indicates the co‐existence of crystalline and amorphous phases in the structure. A slight peak shift toward higher angles and reduction of peak intensities were observed in the Ag‐doped GC.^23^, ^24^, ^25^ Field emission scanning electron microscopy (SEM) images of the pristine and Ag‐doped GC powders are shown in Figure 2c. Both powders appeared as spherical nanoscopic agglomerates. The particle size range for the pristine GC powder was 60–180 nm with a mean size of 80 nm. Silver doping reduced the particle size range to 20–80 nm, with a mean particle size of 36 nm. Energy‐dispersive x‐ray analysis confirmed the presence of Si, Na, Ca, and P in both powder samples. Silver was additionally identified in the Ag‐doped GC powder.

### 2.2 Structural characterization of scaffolds

The nanofibrous scaffolds were fabricated via electrospinning (Figure 1b). The presence of GC and Ag/GC within the nanofibrous scaffolds were evaluated to ensure the fabrication of the proper scaffolds for skin regeneration.

Figure 2d shows the FT‐IR spectra of the gelatin and chitosan (Ch)/polyethylene oxide (PEO)/Gel and the Ag/GC‐Ch/PEO/Gel electrospun scaffolds. The similarity of the two spectra suggests the presence of similar chemical bonds in the two nanofibrous scaffolds. The C=O band at 1662 cm^−1^ was attributed to type I amide. The N—H and C—H bands at 1546 cm^−1^ were attributed to type II amide. The C—N and N—H bands at 1224 cm^−1^ corresponded to type III amide. The N—H vibration at 3298 cm^−1^ was associated with amide groups. These peaks were indicative of the presence of gelatin and chitosan in the scaffolds.^26^, ^27^ The total porosity and pore distribution of the Ag/GC‐Ch/PEO/Gel scaffold were investigated using mercury porosimetry. The results showed that the porosity distribution in this nanofibrous scaffold was in the range of 0.01–20 μm. The average pore size is 7 ± 2 μm (Figure 2e). Since pore size distribution for the Ag/GC‐Ch/PEO/Gel scaffold was the same when evaluated by mercury porosimetry and SEM, the porosity of the other samples was estimated by SEM to be 44%, 39%, and 38% for the Ch/PEO/Gel, GC‐Ch/PEO/Gel and Ag/GC‐Ch/Gel scaffolds, respectively.

Scanning electron microscopy revealed the porous fibrillar structure of the three types of scaffolds (Figure 2f). The mean diameter of the electrospun fibers was 355, 242, and 326 nm for the Ch/PEO/Gel, GC‐Ch/PEO/Gel, and Ag/GC‐Ch/PEO/Gel scaffolds, respectively. The GC‐Ch/PEO/Gel scaffold fibers were considerably finer compared with the other two scaffolds. The incorporation of the GC and Ag/GC powder had no adverse impact on the morphology of the nanofibers. The elemental analysis confirmed the uniform distribution of the GC and Ag/GC powders within the scaffolds.

**FIGURE 3:**
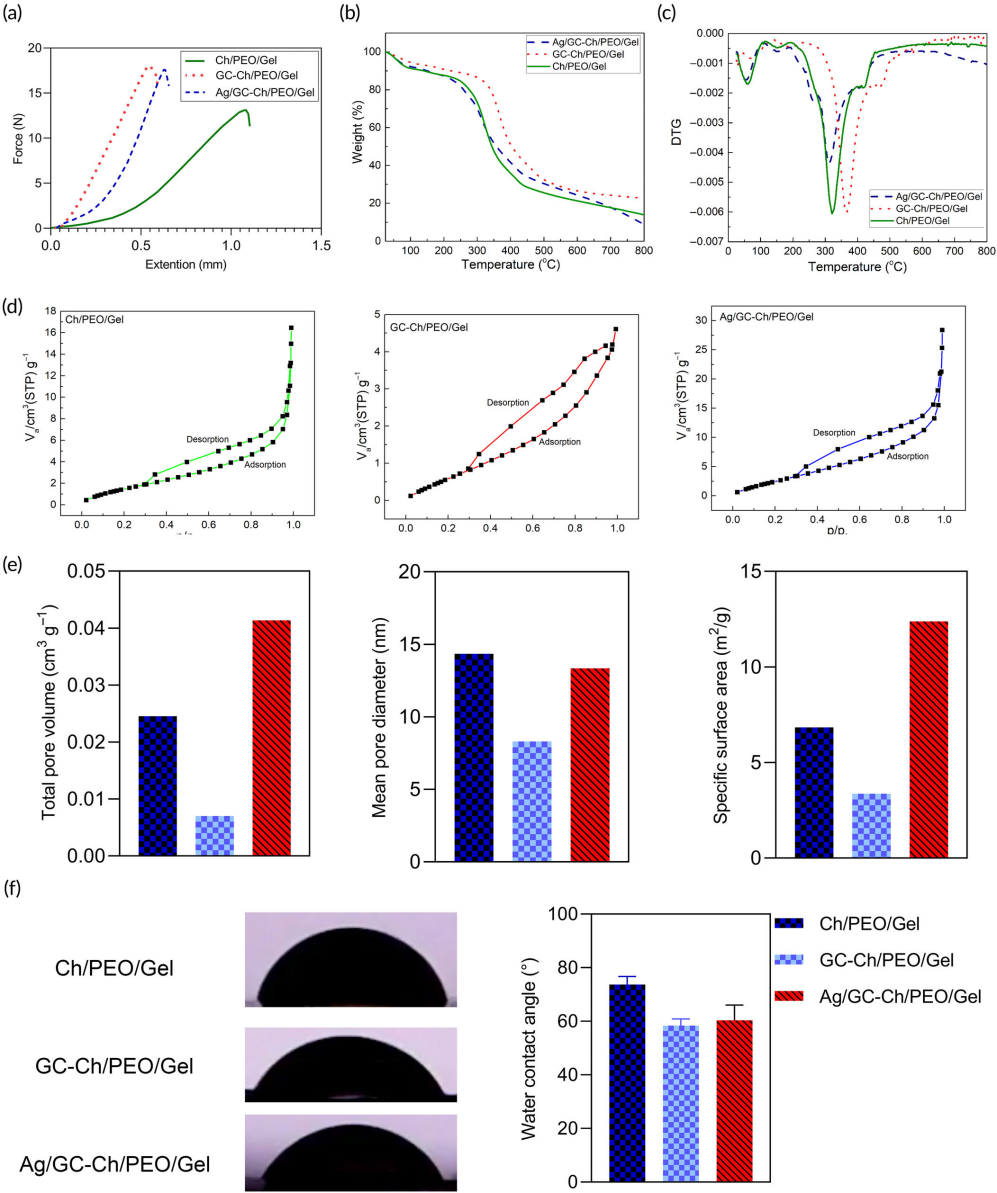
(a) The mechanical behavior of tensile strength for the fabricated nanofibrous scaffolds. TG (b) and DTG (c) of thermograms of Ch/PEO/Gel, GC‐Ch/PEO/Gel, and the Ag/GC‐Ch/PEO/Gel nanofibrous scaffolds. (d) N_2_ adsorption/desorption isotherms of the nanofibrous scaffold samples. (e) The total pore volume, mean pore diameter, and specific surface area of Ch/PEO/Gel, GC‐Ch/PEO/Gel, and Ag/GC‐Ch/PEO/Gel. (f) Water contact angle photos and graph of Ch/PEO/Gel, GC‐Ch/PEO/Gel, and Ag/GC‐Ch/PEO/Gel nanofibrous scaffolds

The mechanical properties of fabricated scaffolds were evaluated using a uniaxial tensile test. The results indicated that the incorporation of GC and Ag/GC improves the Youngs Modulus of the nanofibrous scaffold. However, there was a slight difference between GC‐Ch/GC/PEO/Gel and Ag/GC‐Ch/GC/PEO/Gel groups. Furthermore, the findings demonstrated that the tensile strain at break of GC‐Ch/PEO/Gel and Ag/GC‐Ch/PEO/Gel nanofibrous scaffolds enhanced, compared to Ch/PEO/Gel nanofibrous scaffold, showing that the nanocomposites possess higher mechanical properties than neat nanofibers (Figure 3a). This can be related to the impairing effect of particles such as GCs on the structure of the polymeric scaffolds by serving as hard inclusions.^28^, ^29^

Thermal gravimetric analysis (TGA) measures the weight change of a sample as a function of temperature, while subjected to a controlled heating program. Figure 3b,c show TG and DTG thermograms of Ch/PEO/Gel, GC‐Ch/PEO/Gel, and the Ag/GC‐Ch/PEO/Gel samples. The thermogram comparison of the samples exhibits that the thermal stability of GC‐Ch/PEO/Gel is higher than the Ch/PEO/Gel. Surprisingly, it was observed that the thermal stability of Ag/GC‐Ch/PEO/Gel is lower than the Ch/PEO/Gel. This may be due to the nonoptimized amount of silver in the GC structure.

Brunauer–Emmett–Teller (BET) analysis was used to explain the physical adsorption of gas molecules on a solid surface and serves as the basis for an important analysis technique for the measurement of the specific surface area of materials. Figure 3d show the N_2_ adsorption/desorption isotherms and the BET obtained data of Ag/GC‐Ch/PEO/Gel, GC‐Ch/PEO/Gel, and Ch/PEO/Gel. The BET analysis showed that the specific surface area of Ag/GC‐Ch/PEO/Gel was 12.396 m^2^/g compared to GC‐Ch/PEO/Gel and Ch/PEO/Gel. This can be related to the presence of Ag nanoparticles in the GC composition. Moreover, the mean pore diameter of Ag/GC‐Ch/PEO/Gel was 13.357 nm compared with Ch/PEO/Gel (Figure 3e).

### 2.3 In vitro studies

#### 2.3.1 Contact angle measurement

The water contact angle of the nanofibers was measured to evaluate the wettability of the scaffolds. The Ch/PEO/Gel nanofibrous scaffold had a contact angle of 73.6 ± 3.0°, while GC‐Ch/PEO/Gel and Ag/GC‐Ch/PEO/Gel nanofibrous scaffolds possessed a contact angle of 58.3 ± 2.5° and 60.3 ± 5.6°, respectively. This difference indicated that GC‐containing nanofibers showed higher hydrophilicity (Figure 3f).

#### 2.3.2 Antibacterial activities

**FIGURE 4:**
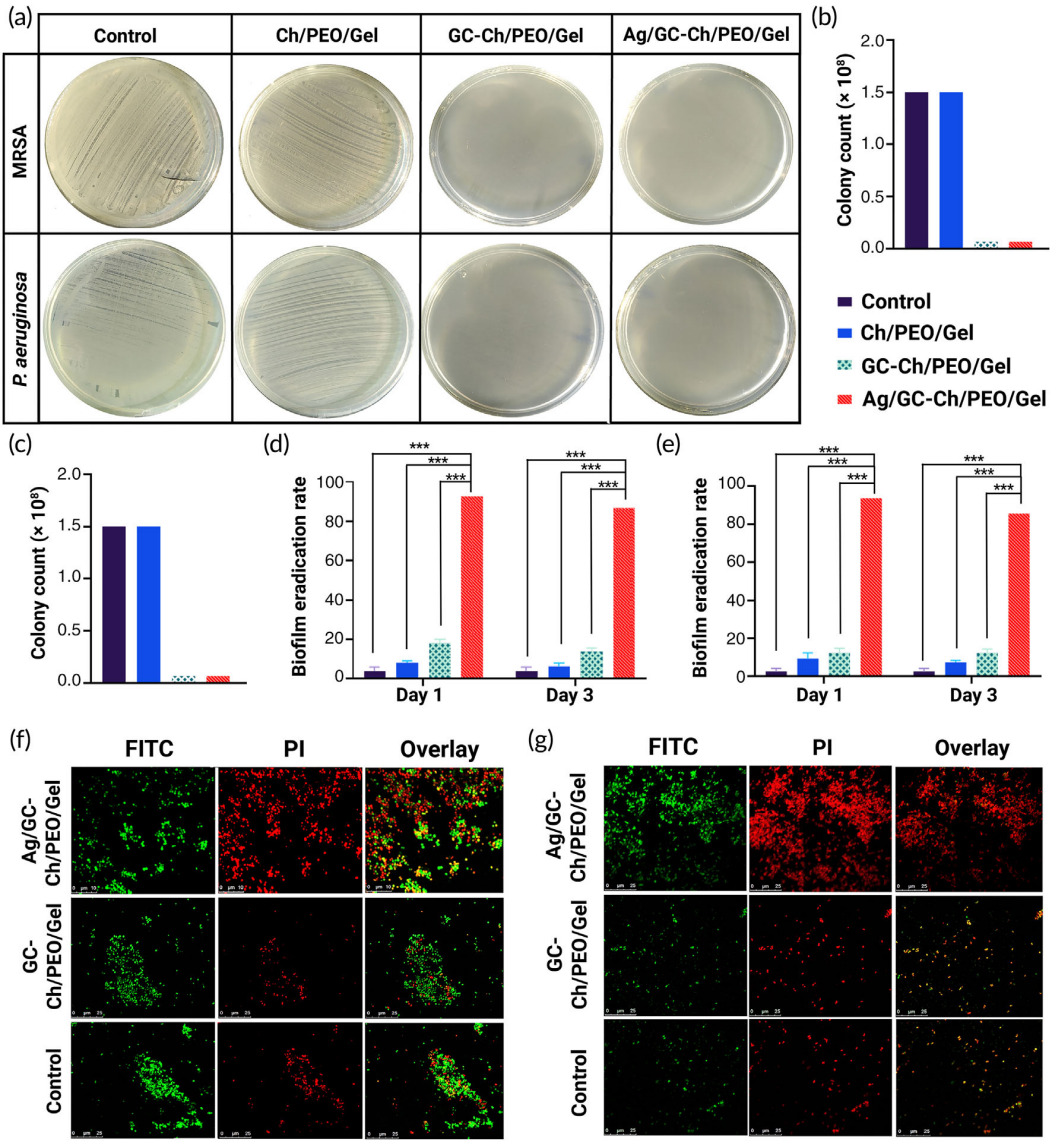
(a) Modified AATCC‐100 Test. After 24 h, no bacterial colony was formed on the media treated with GC‐Ch/PEO/Gel and Ag/GC‐Ch/PEO/Gel suspensions. (b, c) Modified AATCC‐100 Test colony count. After 24 h of exposure of the scaffolds to the bacteria suspension at 37°C, the bacterial colonies were counted and compared with the control group. (d, e) The rate of biofilm eradication of the scaffold suspensions—MRSA (d) and *P*. *aeruginosa* (e). The most effective bacterial eradication was detected when the biofilms were exposed to Ag/GC‐Ch/PEO/Gel (*p* < 0.001). Confocal laser scanning microscopy of 48 h old biofilms of MRSA (f) and *P*. *aeruginosa* (g). After incubation for 24 h, the bacteria were stained with fluorescein isothiocyanate and propidium iodide to evaluate morbidity (green fluorescence—live bacteria; red fluorescence—dead bacteria; f and g). Scale bars represent 25 μm

The antibacterial properties of the nanofibrous scaffolds, with or without GC powder, were investigated using methicillin‐resistant *Staphylococcus aureus* (MRSA) and *Pseudomonas aeruginosa*. Considerable antibacterial activities were identified for the nanofibrous scaffold containing GC powder (Figure 4a). Compared with the control group (1/5 × 10^8^ colony‐forming units [CFU]), there was a significant reduction in CFU (8 logs) after the bacteria were exposed to the scaffolds containing GC nanoparticles (GC‐Ch/PEO/Gel, Ag/GC‐Ch/PEO/Gel nanofibrous scaffolds; Figure 4b,c). As shown in Figure 4d,E, suspensions of the Ag/GC‐Ch/PEO/Gel scaffold had the highest biofilm eradication rate (89 ± 5%). The percentage of biofilm elimination by the Ag/GC‐Ch/PEO/Gel scaffold increased from less than 20% to more than 80% (*p* < 0.001). Confocal laser scanning microscopy of live/dead‐stained single‐species bacteria biofilms showed dead MRSA biofilms (Figure 4f) and *P*. *aeruginosa* biofilms (Figure 4g) after exposure to the AG/GC‐Ch/PEO/Gel scaffolds (red fluorescence). In contrast, biofilms derived from the control group and those exposed to the GC‐Ch/PEO/Gel scaffolds were predominantly alive (green fluorescence).

#### 2.3.3 Cell attachment, biocompatibility, and hemocompatibility

**FIGURE 5:**
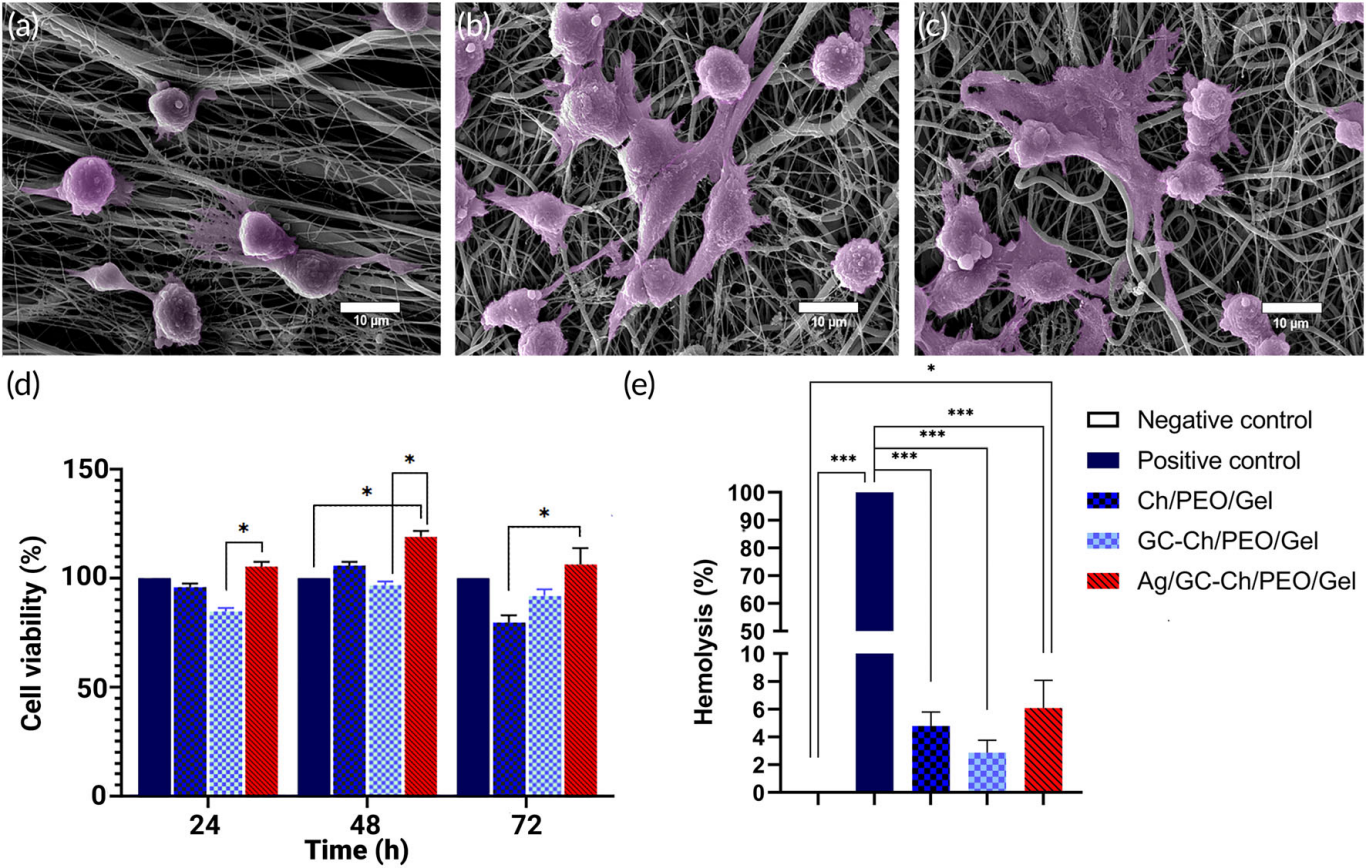
SEM of fibroblasts cultured for 24 h on (a) Ch/PEO/gel, (b) GC‐Ch/PEO/gel, and (c) Ag/GC‐Ch/gel scaffolds. (d) In vitro cytotoxicity of the three experimental scaffolds using the MTT assay with mouse embryonic fibroblasts at 24, 48, and 96 h (*p < 0.05). (e) Percentage of hemolysis induced by different experimental scaffolds. The positive control was blood treated with water. Data are means ± standard deviations (*n* = 3). Groups connected by lines and labeled with asterisks are significantly different; significant differences were tested by one‐way analysis of variance (ANOVA) and post hoc Tukey's multiple comparison tests (*p < 0.05; ***p < 0.001)

Scanning electron microscopy (Figure 5a–c) shows fully attached and well‐spread fibroblasts on all the scaffolds, with many cells bridging the scaffold fibers. Quantitatively, there were more fibroblasts on the scaffolds containing GC powder than those without GC powder. The fibroblasts on these two scaffolds also exhibited better attachment and spreading. The results indicate the potential of the experimental scaffolds to be used for supporting cell survival and growth.

The biocompatibility of Ch/PEO/Gel, GC‐Ch/PEO/Gel, and Ag/GC‐Ch/Gel scaffolds was evaluated using MEFs. After 24 h, no statistically significant changes were observed in the survival percentage of cells treated with Ch/PEO/Gel, GC‐Ch/PEO/Gel, or AG/GC‐Ch/PEO/Gel compared with the control. After 48 h of treatment, the AG/GC‐Ch/PEO/Gel group showed a statistically significant increase compared to the control (*p* < 0.05). When the studied groups were compared after 24, 48, and 72 h, the cell viability percentage in the group treated with AG/GC‐Ch/PEO/Gel increased significantly compared to the group GC‐Ch/PEO/Gel (*p* < 0.05). This increase was also evident after 72 h when compared with the Ch/PEO/Gel (*p* < 0.05) (Figure 5d).

Hemolysis induced by the nanofibrous scaffolds was measured as an indication of hemocompatibility. The Ch/PEO/Gel nanofibrous scaffolds showed 4.79 ± 1% hemolysis. Measurements for the GC‐Ch/PEO/Gel and Ag/GC‐Ch/PEO/Gel scaffolds were 2.86 ± 0.9% and 6.08 ± 2%, respectively (Figure 5e).

#### 2.3.4 The effects of Ag‐doped bioactive GC nanofibrous scaffolds on dendritic cells

**FIGURE 6:**
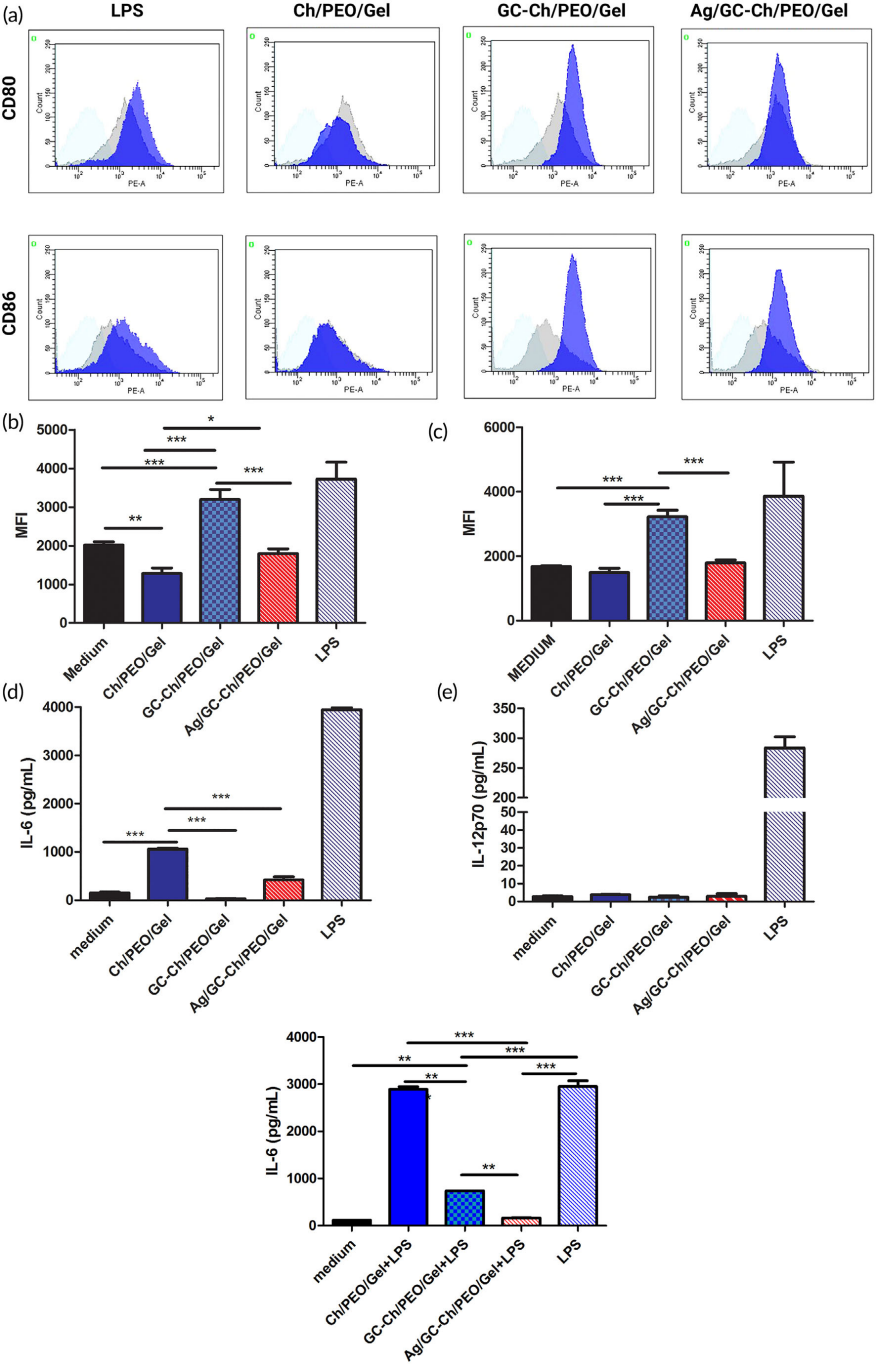
Flow cytometry of immune cell activation markers CD80 and CD86 on bone marrow‐derived dendritic cells that had been exposed to Ch/PEO/Gel; GC‐Ch/PEO/Gel or Ag/GC‐Ch/PEO/Gel scaffolds. Dendritic cells treated with lipopolysaccharides (LPS) were used as the positive control. (a) Mean fluorescence intensity (MFI) of one of the representative experiments. Light blue: isotype control; gray: dendritic cells incubated with medium alone. (b, c) Mean + standard error of the mean of CD80 (b) and CD86 (c) MFI of two independent experiments. Bone marrow‐derived dendritic cells were exposed to the Ch/PEO/Gel, GC‐Ch/PEO/Gel, Ag/GC‐Ch/PEO/Gel scaffolds, or stimulated with lipopolysaccharide (LPS positive control). Culture supernatants were analyzed by enzyme‐linked immunosorbent assay for IL‐6 (d) and IL‐12 p70 (e) production. (f) Inflammation was induced in BM‐DCs by co‐culturing cells with bacterial lipopolysaccharide in the presence of the different scaffolds or the medium. The IL‐6 released in the supernatants was measured by ELISA. All the supernatants were assayed in duplicate. Data represent mean ± standard error of the mean. Results of a representative experiment (out of two) are shown. Significant differences were tested by one‐way analysis of variance (ANOVA) and post hoc Tukey's multiple comparison tests (*p < 0.05; **p < 0.01; ***p < 0.001)

To evaluate the immunogenicity of the nanofibrous scaffolds, bone marrow‐derived dendritic cells were analyzed after the cells were exposed to the different scaffolds. Specifically, the levels of cell surface activation markers CD80 and CD86 were evaluated by cytofluorimetry. The expression of both CD80 and CD86 markers in dendritic cells that had been exposed to the Ch/PEO/Gel scaffold was largely unchanged. Conversely, both markers were upregulated in dendritic cells that had been exposed to the GC‐Ch/PEO/Gel scaffold. Dendritic cells that had been exposed to the Ag/GC‐Ch/PEO/Gel scaffold showed no difference in the levels of CD80 and CD86 expression when compared to untreated dendritic cells (Figure 6a–c).

The release of pro‐inflammatory cytokines interleukin‐6 (IL‐6) and IL‐12 by the dendritic cells was also evaluated. Both IL‐6 and IL‐12 are produced by dendritic cells and macrophages in response to bacterial infections or stress conditions such as tissue injury. The cytokines promote a T helper type I‐oriented response.^30^, ^31^ There was a release of IL‐6 in the dendritic cell cultures that were exposed to the Ch/PEO/Gel scaffold, while the Ag/GC‐Ch/PEO/Gel and GC‐Ch/PEO scaffolds induced slight or no IL‐6 release (Figure 6d). In addition, no release of IL‐12 p70 was found after the exposure of the dendritic cells to all the experimental scaffolds (Figure 6e), further proving their low immunogenicity. Finally, to analyze the anti‐inflammatory effects of the scaffolds against lipopolysaccharide‐induced inflammation, we induced inflammation in dendritic cells by incubating them with lipopolysaccharide in the presence of Ch/PEO/Gel, GC‐Ch/PEO/Gel, and Ag/GC‐Ch/PEO/Gel. Interestingly, we observed that the incorporation of pristine GC powder or Ag‐doped GC powder significantly reduced the LPS‐induced secretion of IL‐6 in dendritic cells, hence the showing anti‐inflammatory properties of these two scaffolds (Figure 6f).

### 2.4 In vivo study

**FIGURE 7:**
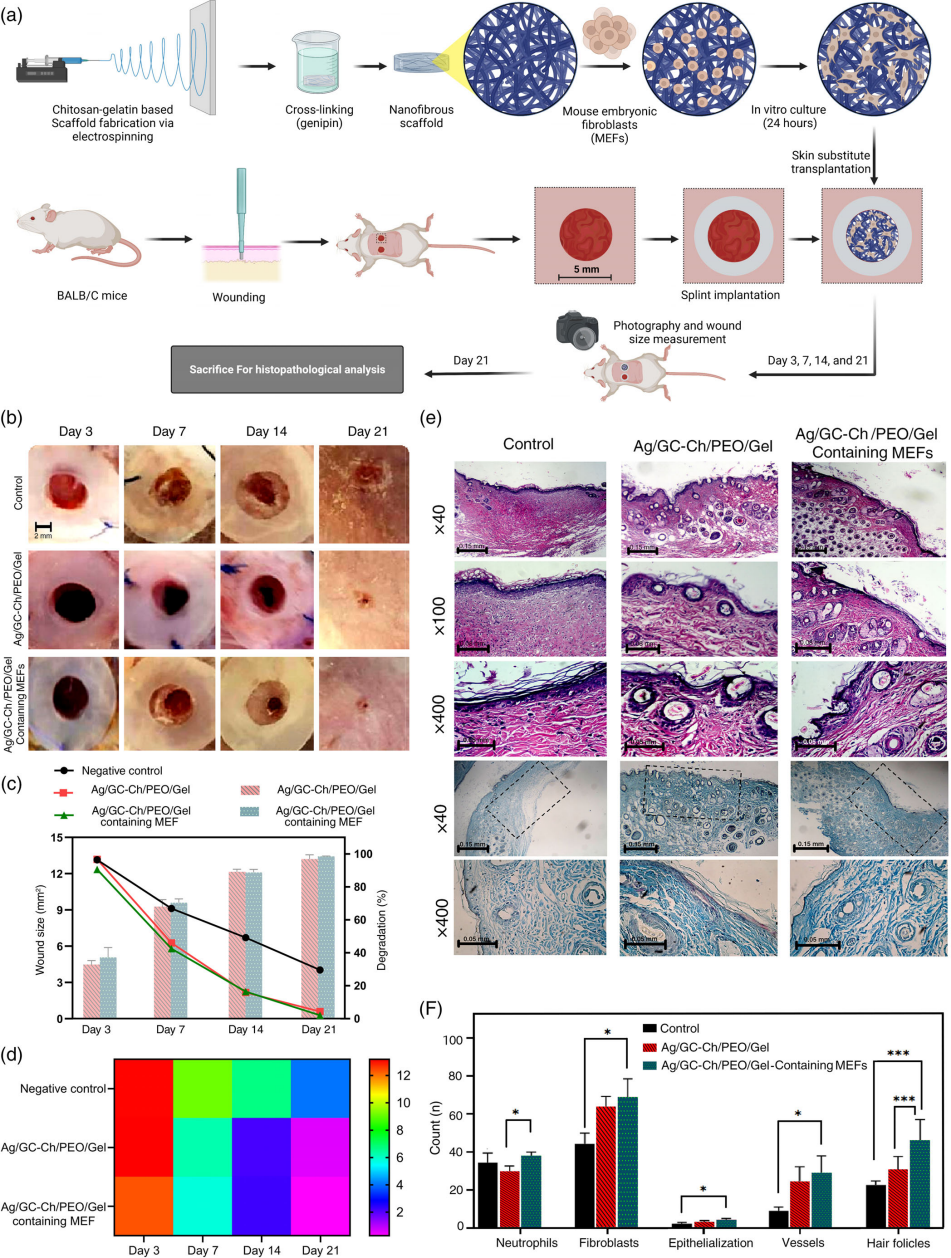
(a) Schematic illustration of the procedure for fabrication of scaffold and in vivo study. (b) Macroscopic wound healing of full‐thickness excisional wound model on the back of the BALB/c mice up to 21 days. (c) Wound closure based on the size of the wound relative to the original dimension of the damage. Biodegradation percentage of Ag/GC‐Ch/PEO/Gel nanofibrous scaffolds according to remaining scaffolds on the wound. Results are the means of three measurements. SD was always lower than 10%. Error bars were omitted for graph clarity. (d) The heatmap diagram display changes in wound dimension with time over 3 weeks. (e) H&E staining on the 21st day; and Masson's trichrome staining on the 21st day. (f) The average count of neutrophils, fibroblasts, hair follicles, epithelium layers, and blood vessels after treatment of the wounds with Ag/GC‐Ch/PEO/Gel scaffolds with/without MEFs compared to the control (data are means and standard deviations; *p < 0.05; ***p < 0.001)

The wound healing efficacy of the Ag/GC‐Ch/PEO/Gel nanofibrous scaffold, with or without MEFs, was evaluated using a BALB/c mouse excisional wound splinting model and followed histopathological analysis. As shown in Figure 7b,c, the size of all the wounds was approximately equal to the one on the third day. Wounds size treated with the Ag/GC‐Ch/PEO/Gel nanofibrous scaffold, with or without MEFs, became considerably smaller in dimension than the control group after the third day. In comparison, wound size in the control wound area remained the same, with minimal change (9.12 ± 0.85 mm^2^). On the seventh day after surgery, wound healing and skin regeneration in the Ag/GC‐Ch/PEO/Gel and the Ag/GC‐Ch/PEO/Gel containing MEFs improved significantly (6.27 ± 0.83 and 5.80 ± 0.45 mm^2^) compared with the control group (9.12 ± 0.85 mm^2^). Investigations on the 14th day revealed the same healing process in wounds that were treated by the nanofibrous scaffold; there was a lack of skin regeneration in the control group. Compared with other groups (4.03 ± 0.17 mm^2^) and after 21 days, there was a significant decrease in wound area in those skin wounds that were treated with Ag/GC‐Ch/PEO/Gel scaffolds containing MEFs (0.28 ± 0.04 mm^2^). According to the macroscopic evaluation of wound healing (Figure 6b) as well as wound size (Figure 6c), the rate of biodegradation of the Ag/GC‐Ch/PEO/Gel nanofibrous scaffold was equivalent to the rate of skin regeneration.

The histopathology results are shown in Figures 7e,f. The epidermal layer was incompletely regenerated in the negative control group; inflammatory cell infiltration was apparent in the dermis. The lowest epithelialization and collagen synthesis were observed in the negative control group. Conversely, treatment of the wounds with the Ag/GC‐Ch/PEO/Gel and Ag/GC‐Ch/PEO/Gel nanofibrous scaffolds containing MEFs induced thicker epidermal layer formation, more profuse epithelialization, collagen synthesis, and angiogenesis. Epithelialization was complete in the wounds treated with the Ag/GC‐Ch/PEO/Gel nanofibrous scaffolds with or without MEFs.

The skin appendix was completely regenerated using the Ag/GC‐Ch/PEO/Gel nanofibrous scaffold containing MEFs. The full skin layer thickness was observed that contain characteristic rete ridges, sebaceous glands, and hair follicles. Based on previous studies on wound regeneration,^32^, ^33^ increases in fibroblast and hair follicle counts could be attributed to the GCs. More precisely, there were 70 ± 3 and 75 ± 6 fibroblasts, 30 ± 5 and 75 ± 7 hair follicles when skin wounds were treated with the Ag/GC‐Ch/PEO/Gel nanofibrous scaffold and the Ag/GC‐Ch/PEO/Gel nanofibrous scaffold containing MEFs, respectively. In contrast, the fibroblast count for the control group was 43 ± 4 fibroblasts. For hair follicles, there were 23 ± 2 follicles in the control group, and 32 ± 7 and 44 ± 9 follicles in the wounds treated by the Ag/GC‐Ch/PEO/Gel nanofibrous scaffold and Ag/GC‐Ch/PEO/Gel nanofibrous scaffold containing MEFs, respectively. The hypodermis is the lowest skin layer that acts as an insulator. There were more prominent blood vessels in the hypodermis in wounds that were treated with the Ag/GC‐Ch/PEO/ Gel nanofibrous scaffold containing MEFs. In comparison, regeneration was incomplete in the control and Ag/GC‐Ch/PEO/Gel groups. In the second skin layer (i.e., the dermis), more elastin fibers were identified from wounds treated with scaffolds containing MEFs. In some wounds treated by the Ag/GC‐Ch/PEO/Gel nanofibrous scaffold containing MEFs, neutrophils with the N2 phenotype could be observed.

The outermost skin layer (i.e., the epidermis) was not regenerated in the control group. Conversely, the epidermis was healed entirely in wounds that were treated with the Ag/GC‐Ch/PEO/Gel nanofibrous scaffold. Regeneration of the epidermis was attributed to keratinocytes, which are mostly presented in skin treated with the Ag/GC‐Ch/PEO/Gel nanofibrous scaffold containing MEFs. Examination of the skin layer thickness using Masson's trichrome staining revealed that wounds treated with the Ag/GC‐Ch/PEO/Gel nanofibrous scaffold containing MEFs had the narrowest fibrosis layer. This indicates that regenerated skin treated with the Ag/GC‐Ch/PEO/Gel nanofibrous scaffold containing MEFs had the highest collagen density and degree of angiogenesis. Wounds treated with the Ag/GC‐Ch/PEO/Gel with MEFs contained 30 ± 5 blood vessels. Wounds treated with the Ag/GC‐Ch/PEO/Gel contained 24 ± 6 vessels. In stark contrast, there were only 9 ± 2 vessels in wounds derived from the control group.

## 3 DISCUSSION

Regenerative medicine and tissue engineering are promising therapeutic approaches for repairing or replacing large skin wounds. An extensive array of biomaterials, semiconducting nanomaterials, bioactive glass, GC, and composite materials are available to develop skin substitutes.^34^ Skin substitutes with angiogenic and antibacterial properties and the ability for tissue to regenerate are ideal alternatives to traditional dressings because they improve the wound healing process.^23^, ^35^

In the present work, GC‐Ch/PEO/Gel nanofibrous scaffolds containing pristine GC or Ag‐doped GC were synthesized. To enhance cellular interactions, 60–80 nm sol–gel derived GC or Ag/GC were incorporated into the electrospun scaffolds that had fibrils with diameters between 200 and 300 nm. The electrospinning setting employed produced Ag/GC‐Ch/PEO/Gel scaffolds with a mean pore size of 7 ± 2 μm. A recent study reported that the optimal pore size for cellular infiltration and vessel formation in electrospun nonwoven scaffolds for bioresorbable vascular grafts is in the range of 5–20 μm.^36^

X‐ray diffraction showed that the GC powder had combeite as the predominant crystalline phase. This result was in agreement with the literature.^25^, ^37^, ^38^, ^39^ The reason for the formation of this crystalline structure is that the temperature at which nitrates are completely removed is higher than the crystallization temperature of the glass. A lower degree of crystallinity was identified for Ag‐GC, making the glass–ceramic more suitable for biological applications. Fourier transform‐infrared spectroscopy confirmed that both the GC and Ag/GC powders possessed 45S5 bioglass characteristics. Energy‐dispersive ‐ray analysis confirmed the presence of Ag in the Ag‐doped GC powder. Infrared spectra of the scaffolds also identified the presence of gelatin (amide types I, II, III, and amide B), chitosan, PEO, and GC in the nanofibrous scaffold network.

Cell–scaffold interactions are significantly affected by the surface's wettability of the scaffolds, as this feature controls several key biological processes, including protein adsorption, cell attachment, and proliferation.^11^, ^40^ Furthermore, the contact angle is a proper assay to measure the surface moisture, which plays a vital role in wound healing.^41^ The difference indicates that GC and Ag/GC containing nanofiber scaffolds possess higher hydrophilicity. The presence of GC particles on the fiber surface results in a rougher and more hydrophile surface, which improves the wettability of these composite fibers.^42^, ^43^ Surfaces with a contact angle of fewer than 90° are hydrophilic, while those with a contact angle higher than 90° are hydrophobic. Biomaterials having excessively hydrophobic or hydrophilic surfaces are not suitable for cell adhesion. Hydrophilicity in the normal range allows proper attachment of protein and cell on the surface. Furthermore, wound dressings that are hydrophilic and deliver moisture to the wound site can accelerate and ameliorate wound healing.^40^, ^44^

**FIGURE 8:**
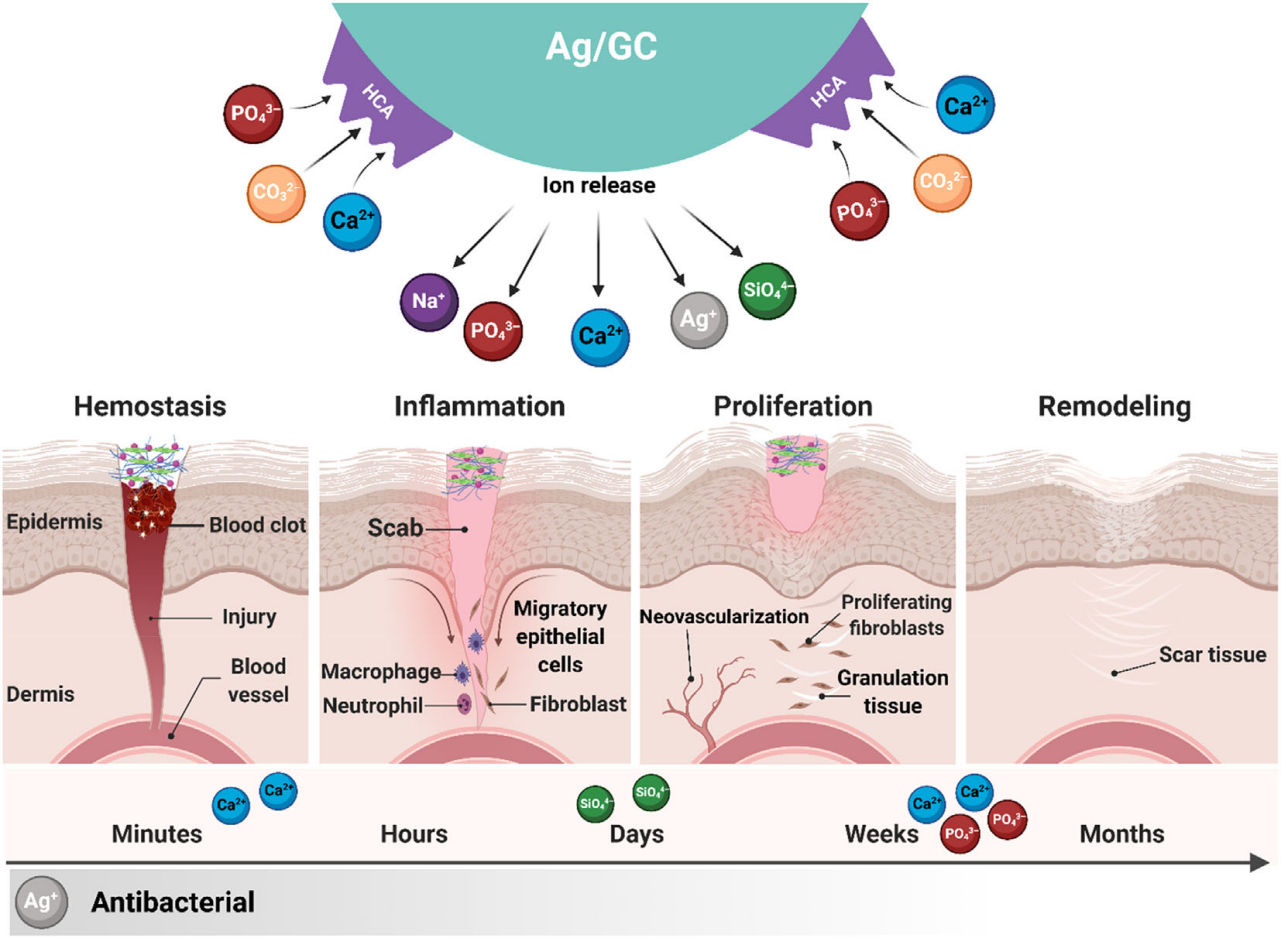
Schematic of the wound healing process and the effect of the release of various ions. Ion release from nanoparticles upon exposure to biological fluid and their potential effect in different stages of wound healing

The GC‐Ch/PEO/Gel and AG/GC‐Ch/PEO/Gel nanofibrous scaffolds demonstrated potent antibacterial activities against Gram‐positive and Gram‐negative bacteria. During the interaction of GC nanoparticles with body fluids, ions such as Na^+^ and Ca^2+^ are exchanged with H^+^/H_3_O^+^ ions. An increase in pH due to the release of the cations and an increase in osmotic pressure caused by the formation of phosphorus and calcium salts render the environment unfavorable in terms of bacteria adhesion and proliferation, which substantially reduces the risk of infection (Figure 8).^45^, ^46^ In comparing the antibacterial and anti‐biofilm results, it may be concluded that GC possesses antimicrobial properties but does not eliminate bacterial biofilms. The biofilms were eliminated after they were exposed to Ag ions. The antibacterial experiments also showed that Ag doping in the GC structure augments the intrinsic antibacterial activity of this biomaterial. The nanofibrous scaffold containing Ag‐doped GC powder appears to be a better choice for wound dressing.

The fabricated nanofibrous scaffold exhibited a high degree of biocompatibility and may be used as a tissue engineering skin substitute to expedite the healing of skin wounds.^24^, ^47^ The scaffolds were conducive to cell attachment and bridging of the fibers by cell extensions. The ECM‐like lattice of the scaffolds enables physical interactions with cells and provides a matrix for cells to attach, survive, proliferate, and communicate.^17^, ^48^ The ability of chitosan/gelatin fiber blends to support the survival and proliferation of human dermal fibroblasts has previously been demonstrated.^49^

The hemolysis assay is an essential blood compatibility test for determining a material's biocompatibility. Damaged RBCs release adenosine diphosphate, which increases platelet attraction and assembly to the material surface. This procedure may expedite the initiation of coagulation cascades and thrombosis, leading to disruption of the wound healing process. Accordingly, a desirable skin substitute should not harm the circulating RBCs at the wound site and should not compromise the activation of coagulation pathways.^50^ The results indicate that all the experimental scaffolds examined, the GC‐Ch/PEO/Gel scaffold, in particular, induced negligible hemolysis.

Biocompatible materials should be well‐tolerated by the host. Consequently, they should be immunologically inert and should not induce inflammatory host immune responses.^51^ Results from the present study demonstrate that the materials containing GC powder did not induce any significant inflammatory response. The upregulation of dendritic cell markers by the GC‐Ch/PEO/Gel scaffold suggests that this material can induce a certain degree of dendritic cell maturation without releasing inflammatory mediators, with the switch to a more anti‐inflammatory phenotype. Moreover, a co‐culturing study of DCs and the different scaffolds in the presence of bacterial lipopolysaccharide showed that the nanofibers containing GC or Ag/GC can suppress the effect of LPS‐induced inflammation. These findings demonstrate that the scaffolds containing GC powder do not elicit a significant immune response and are suitable for wound healing applications.

Histopathological analysis showed that wounds treated with the Ag/GC‐Ch/PEO/Gel nanofibrous scaffold were in the maturation phase of healing with negligible inflammation. In contrast, wounds in the negative control had undergone fibroplasia due to the integration of collagen and stimulation of angiogenesis. Polygonal cells separated by narrow, translucent clefts located in stratum spinosum could be recognized. Examination of hematoxylin and eosin‐stained and Masson's trichrome‐stained sections of the healed wounds in vivo confirmed better regeneration of the damaged skin in wounds grafted with the Ag/GC‐Ch/PEO/Gel nanofibrous scaffold containing MEFs. This is attributed to better deposition and connection of the collagen fibrillar network. Although there is some angiogenesis in the negative control, the enhanced angiogenesis observed in the wounds treated with the Ag/GC‐Ch/PEO/Gel nanofibrous scaffold, with or without MEFs, may be attributed to the bioactivity of the GC powder. The secretion of angiogenic factors probably causes an increased number of regenerated blood vessels under the influence of the GC powder.

The results indicate that wounds were regenerated without scar tissue formation in the presence of Ag/GC‐Ch/PEO/Gel nanofibrous scaffold containing MEFs. Wound healing in adult mammals generally results in scar tissue that lacks skin appendages such as hair follicles and sebaceous glands. Accordingly, complete tissue regeneration is a challenge. While scar development may fulfill the primary role of the skin in preventing infection and dehydration, it may also be unfavorable. The scar formed as a result of injuries or burns can have severe aesthetic and psychological consequences, affecting the individual's quality of life due to its noticeable difference in appearance from the original intact skin. As scar tissue formation interrupts the complete skin recovery, the potential to regenerate the skin to its natural condition is highly valued.^52^, ^53^

## 4 MATERIALS AND METHODS

### 4.1 Materials

The materials used in the present work include tetraethyl orthosilicate (TEOS, MilliporeSigma, Burlington, MA), triethyl phosphate (TEP, Merck, Kenilworth, NJ), nitric acid (HNO_3_), calcium nitrate tetrahydrate (Ca[NO_3_]_2_·4H_2_O), sodium nitrate (NaNO_3_), silver nitrate (AgNO_3_) and potassium bromide (KBr, IR grade) were used for the synthesis of GC and Ag/GC nanoparticles as well as structural characterization. Gelatin (MilliporeSigma), chitosan (deacetylated ≥90%, viscosity 20–500 mPa, Solarbio Life Science, Beijing, China), polyethylene oxide (PEO, MW: 900 kDa, MilliporeSigma), acetic acid (CH₃COOH), genipin (C_11_H_14_O_5_) were used for synthesizing the matrix of the nanofibrous scaffolds. Mueller–Hinton agar was used for antibacterial evaluation. 2,3,5‐Triphenyl‐tetrazolium chloride (TTC, MilliporeSigma) and tryptic soy broth were used for TTC assay. Phosphate‐buffered saline (PBS), propidium iodide (MilliporeSigma), and fluorescein isothiocyanate (MilliporeSigma) were used for confocal laser scanning microscopy. 3‐(4, 5‐dimethylthiazol‐2‐yl)‐2, 5‐diphenyl tetrazolium bromide (MTT), DMSO, ethanol, and glutaraldehyde were used for cell viability and cell attachment measurement. MEF cells were obtained from the National Cell Bank of Iran. C57BL/6 mice (Charles River, Lecco, Italy), RPMI 1640 medium, recombinant murine granulocyte/macrophage colony‐stimulating factor (GM‐CSF, Peprotech, Rocky Hill, NJ), fetal bovine serum (FBS), penicillin, streptomycin, sodium pyruvate, 2‐mercaptoethanol, and anti‐CD11c‐PE‐Cy7 (HL3, BD Biosciences, Franklin Lakes, NJ) were utilized for phenotypic characterization of BM‐DCs and cytokine production. Cefazolin, ketamine/xylazine, povidone‐iodine, Vicryl® sutures, buffered were utilized for skin wound creation. Buffered formalin (10%, pH:7.26); paraffin, hematoxylin & eosin (H&E), and Masson's trichrome were used for histopathological evaluations.

### 4.2 Synthesis and structural characterization of GC and Ag/GC nanoparticles

Two types of GC nanoparticles, pristine and Ag‐doped, were synthesized using the sol–gel method. The GC has a 45S5 bioactive glass composition: silica (SiO_2_, 45% wt), calcium oxide (CaO, 24.5% wt), sodium oxide (Na_2_O, 24.5% wt), and phosphorous pentoxide (P_2_O_5,_ 6% wt).^20^ Briefly, 33.5 ml of tetraethyl orthosilicate was added to 50 ml of 1 M HNO_3_ for hydrolysis under continuous stirring for 60 min. This step was followed by adding 2.9 ml of triethyl phosphate, 20.13 g of calcium nitrate tetrahydrate, and 13.52 g of sodium nitrate. For Ag/GC synthesis, Ag was partially substituted for Na in the network. For this purpose, 20.13 g of calcium nitrate tetrahydrate, 12.96 g of sodium nitrate, and 0.48 g of silver nitrate were added to the precursor solution in three steps with a time interval of 45 min under continuous stirring. The prepared solutions were kept in a sealed cylindrical Teflon container at room temperature for 2 days until the gels were formed. The wet gels were dried at 120°C in an oven for 16 h to produce xerogels. The resultant powders were transferred to a furnace and sintered at 700°C to remove nitrates and stabilize the network.^54^ Grinding of the sintered GC powder was performed using a ball mill to obtain nanoparticles (Figure 1a).

Structural characterization of the synthesized GC was conducted with a Philips X‐ray diffractometer (XRD) with Cu‐*K*α radiation (λ = 1.78901 Å) with a step size of 0.02°, scanning time of 1 s, and scanning range of 5°–70°. The size, morphology, and chemical composition of the GC were evaluated using field‐emission scanning electron microscopy (FESEM, MIRA3 TESCAN‐XMU, Tescan, Brno, Czech Republic) equipped with energy dispersive spectrometry (EDS). Fourier transform‐infrared spectroscopy (FT‐IR, Nicolet 800, Nicolet Instrument Corp., Madison, WI) was used to identify the functional groups of the prepared GCs. For this purpose, 1 mg of each powder was mixed with 300 mg of KBr, prepared as a pellet, and analyzed over the range of 4000–400 cm^−1^ at a scanning speed of 2.60 Hz with a resolution of 4 cm^−1^.

### 4.3 Fabrication and structural characterization of nanofibrous scaffolds

Three types of nanofibrous scaffolds (Ch/PEO/Gel, GC‐Ch/PEO/Gel, and Ag/GC‐Ch/PEO/Gel) were fabricated through electrospinning. Briefly, 18% w/v gelatin and 2% w/v chitosan were dissolved in 80% v/v acetic acid and added to 3.5% w/v of PEO dissolved in 0.5 M acetic acid. The resultant solution was mixed with 1% wt of either GC or Ag/GC powder and stirred at room temperature overnight. The suspension was then loaded into a syringe. Electrospinning was performed at ambient temperature under the following conditions; voltage: 20 kV, distance: 150 mm, injection rate: 1 ml/h. Crosslinking with 1% genipin for 4 h was used to enhance the mechanical properties of the nanofibrous scaffolds. Disc‐like scaffolds with a diameter of 5 mm were prepared by punching for further applications (Figure 1b).

Morphological and semi‐quantitative elemental analyses of the prepared nanofibrous scaffolds were conducted using FESEM at an accelerating voltage of 20 kV. The nanofibers were sputter‐coated with gold before examination. The diameter of the nanofibers was measured using Image J (1.47v, National Institute of Health, Bethesda, MD) software.

Fourier transform‐infrared spectroscopy was used to identify the functional groups of the prepared nanofibrous scaffolds. Briefly, 1 mg of each scaffold was mixed with 300 mg of KBr. The mixture was prepared as a pellet and analyzed over the range of 4000–400 cm^−1^ at a scanning speed of 2.60 Hz with a resolution of 4 cm^−1^.

Total porosity, average pore diameter, and pore size distribution of the Ag/GC‐Ch/Gel scaffolds were evaluated using mercury porosimetry (PASCAL 140, Thermo‐Finnigan LLC, San Jose, CA) using increasing pressures of 0.1–400 kPa. Pore size measurements were performed on the SEM micrographs of the prepared nanofibrous scaffolds using the ImageJ software.

The mechanical characteristics of the nanofibrous scaffolds were measured using SANTAM universal testing machine (STM‐1 model). Specimens were cut into 80 × 15 mm with a 400 ± 20 μm diameter. The ends of the rectangular specimens were placed vertically on the tensile tester's two mechanical gripping components, leaving 50 mm gauge length for mechanical loading, and were pulled with a 5 mm/min rate.

Thermogravimetric analysis (TGA, L81A1750, Linseis) was employed to study the thermal stability of fabricated nanofibrous scaffolds. TGA analysis was recorded at 10°C/min in an N_2_ atmosphere.

N_2_ adsorption–desorption isotherms were obtained on a Nova 2000 pore analyzer at 196°C under continuous adsorption condition. Brunauer–Emmett–Teller (BET) analyses were utilized to determine the surface area, the pore size distribution and the pore volume.

### 4.4 In vitro studies

#### 4.4.1 Contact angle measurement

The hydrophobic characteristics of each specimen were assessed using contact angle measurement by 2X lens and Protractor (AMCAP, VERSION 9.016). Nanofibrous scaffold samples were prepared into 12 × 12 mm^2^ square pieces and fixed on the assay plate. Afterward, a single drop with an approximate volume of 4 μl of distilled water was added to each sample at room temperature. Three different areas on each sample were measured, and the mean contact angle and standard deviation were calculated.

#### 4.4.2 Antibacterial evaluation

The bacterial death rate was evaluated using a modified AATCC‐100 Test Method. The bacterial death rate was measured by providing 4 cm^2^ of nanofibrous scaffolds and a bacterial suspension prepared in LB broth (*S*. *aureus*, ATCC 25923 and *P*. *aeruginosa*, ATCC 27853, American Type Culture Collection, Manassas VA) containing 1.5 × 10^8^ colony forming units (CFU)/ml (0.5 McFarland). One hundred microliters of the suspension were placed on the scaffolds and sandwiched with other materials. After 24 h of incubation at 37°C, the nanofibrous scaffold and suspension were shaken, and 0.1 ml of each sample was cultured on the Muller Hinton agar medium. Bacteria colonies were counted after 24 h of incubation at 37°C by deploying the colony counter plugin of the ImageJ software bundled with 64‐bit Java 1.8.0_172 (National Institute of Health, Bethesda, MD), which were compared with the bacterial quantities in control groups [1/5 × 108 colony‐forming units (CFU)].^55^

#### 4.4.3 Trimethyl tetrazolium chloride assay

To determine the anti‐biofilm properties of the nanofibrous scaffolds, trimethyl tetrazolium chloride (TTC) was used to detect *P*. *aeruginosa* and MRSA (methicillin‐resistant *Staphyloccocus aureus*) biofilms. A 96‐well cell culture microtiter plate was used to form the biofilms using a bacterial suspension prepared in Tryptic Soy Broth (TSB) containing 1.5 × 10^8^ colony forming units (CFU)/ml (0.5 McFarland). The bacterial suspension was incubated for 24 h at 37°C. The test was performed on the 1‐day and 3‐day biofilms. To treat the biofilms, 4 cm^2^ of a nanofibrous scaffold was placed in the TSB medium on a shaker for 24 h. Planktonic cells were removed from each microtiter plate well and rinsed three times using prewarmed (37°C) physiological saline to observe the anti‐biofilm activity. Then, 200 μl of the scaffold suspension was added to each well. The wells were evacuated after 24 h of incubation at 37°C. The TTC solutions were prepared by dissolving 0.1% TTC in distilled water and filtered using 0.22 μm cellulose acetate filters. A 250 μl of TSB containing 50 μl of TTC solution was added to wells and incubated for 2 h at 37°C and 120 rpm. After the incubation period, 200 μl of the well contents were moved to a new flat‐bottomed microplate. The absorbance was measured at 540 nm (Epoch Microplate Spectrophotometer, BioTek, Winooski, VT).^56^, ^57^

#### 4.4.4 Confocal laser scanning microscopy

Confocal laser scanning microscopy to assess the impact of the GC‐containing nanofibrous scaffold on the MRSA and *P*. *aeruginosa* biofilms. The biofilms were grown on glass coverslips as previously described.^56^ In brief, 6‐well microtiter plates were seeded with glass coverslips. Five milliliters of TSB with 2% glucose were added to each well. Three microliters of mid‐exponential grown bacterial culture in TSB were added aseptically to the wells, followed by incubation at 37°C for 24 h. Scaffold suspensions were then added to the wells except for the control wells. The culture plates were incubated at 37°C for 24 h. The coverslips were taken out and softly rinsed with sterile PBS and stained with 15 μl propidium iodide for 15 min at room temperature to identify dead bacterial cells. To visualize the green glycocalyx matrix, 50 μl/ml of fluorescein isothiocyanate (FITC) was added to the wells for 15 min at room temperature. The propidium iodide and FITC were excited at 540 and 630 nm, respectively. Their emission was separately observed at 490 and 535 nm, respectively. Untouched biofilms were analyzed using CLSM (Leica TCs SP5 ll, Leica Biosystems, Wetzlar, Germany).

#### 4.4.5 Cell viability and cell attachment measurement

For SEM examination of cell attachment, the prepared nanofibrous scaffolds were seeded with 1 × 10 ^4^ MEF for 3 days. This was followed by fixation in 2.5% glutaraldehyde for 1 h and dehydration in a series of sequentially increasing concentrations of ethanol solutions (30%, 50%, 70%, 80%, 90%, and 100%) for 20 min each.^19^

Biocompatibility of the fabricated nanofibrous scaffolds was evaluated using 3‐(4,5‐dimethylthiazol‐2‐yl)‐2, 5‐diphenyl tetrazolium bromide (MTT) assay. After sterilizing the scaffolds by soaking in 70% ethanol followed by 2 h of UV radiation and washing twice with both PBS and cell culture media, MEFs were seeded on the top of the nanofibrous scaffolds at a density of 1 × 10^4^ cells per scaffold. Cell viability for each scaffold was evaluated using the MTT assay. Every 96‐well was filled with 100 μl of MTT solutions (0.5 mg/ml) and incubated at 37°C for 4 h. The content of the wells was removed, and 200 μl of DMSO was added to each well. After 30 min, the absorbance was read using a microplate reader (Stat fax‐2100, Awareness Technologies, Ramsey, MN) at 570–630 nm after 24, 72, and 96 h. The MEF cells in 2D culture served as the control.^58^

#### 4.4.6 Hemocompatibility evaluation

Hemolysis of red blood cells after incubation with the prepared nanofibrous scaffolds was measured as a function of hemocompatibility. Briefly, the samples were incubated for 60 min at 37°C with 200 μl of fresh and anticoagulated blood diluted with PBS. After incubation, the samples were centrifuged for 10 min at 1500 rpm. The absorbance of the supernatant was read at 545 nm using the microplate reader. The percentage of hemolysis was calculated using the formula

$$\text{Hemolysis}\;\left(\%\right)=\frac{\mathrm{Dt}-\mathrm{Dnc}}{\mathrm{Dpc}-\mathrm{Dnc}}\times 100$$

where Dt is the absorbance of the sample, Dnc is the absorbance of the negative control, blood diluted with PBS without any treatment, and Dpc is the absorbance of the positive control.

#### 4.4.7 Generation of bone marrow‐derived dendritic cells

Female C57BL/6 mice (Charles River Laboratories, Lecco, Italy) were used for the generation of bone marrow‐derived dendritic cells (BM‐DCs). The BM‐DCs were derived from precursors isolated from the tibiae of euthanized mice. Briefly, both ends of the tibiae were cut. Bone marrow was flushed with a syringe needle filled with ice‐cold Roswell Park Memorial Institute (RPMI) 1640 medium. After separation of the cell clusters, the cells were washed twice with medium, seeded at a density of 2 × 10^6^ cells per 10 cm dish (Falcon, no. 1029, bacterial quality, Heidelberg, Germany), and cultured with 200 U/ml recombinant murine granulocyte/macrophage colony‐stimulating factor (GM‐CSF, Peprotech) in RPMI 1640 medium supplemented with 10% fetal bovine serum (FBS), 100 U/ml penicillin, 100 μg/ml streptomycin, 1 mM sodium pyruvate and 50 μM 2‐mercaptoethanol. Immature BM‐DCs were collected on the seventh day of culture and were assayed for dendritic cell phenotype by staining with the monoclonal antibody anti‐CD11c‐PE‐Cy7 (HL3, BD Biosciences) and fluorescence‐activated cell sorting (BD FACS Canto II, BD Biosciences).

#### 4.4.8 Phenotypic characterization of BM‐DC and cytokine production

To determine the effect of the nanofibrous scaffolds on the modulation of DC phenotype, 1 × 10^6^/ml BM‐DCs at the seventh day of culture were seeded on the top of the scaffolds in 48‐well plates in RPMI medium, supplemented as described previously. Cultures were also left untreated (medium) or treated with 5 μg/ml of lipopolysaccharides from *Escherichia coli* serotype 055:B5 as the positive control. After 20 h of culture, the cells were recovered, washed twice with PBS, and stained with the following antibodies: anti‐CD11c‐allophycocyanin (HL3), anti‐CD80‐PE (16‐10A1), and anti‐CD86‐PE (GL1). Analysis was performed by flow cytometry using the BD FACS Canto II cytometer and DIVA software.

To analyze the production of IL‐6 and IL‐12p70, supernatants were collected from BM‐DC cultures after they were in contact with the scaffolds for 20 h or with lipopolysaccharide (LPS) as a positive control. In some experiments, BM‐DCs were co‐cultured with bacterial lipopolysaccharide (5 μg/ml) in the presence of the different nanofibrous scaffolds or RPMI complete medium. The IL‐6 or IL‐12p70 in the supernatants (0.1 ml) were measured according to the manufacturer's instructions using commercially available mouse IL‐6 ELISA MAX™ Standard (Biolegend, San Diego, CA) and mouse IL‐12p70 ELISA ready‐SET‐Go ELISA kits (Invitrogen, ThermoFisher Scientific, Waltham, MA), respectively.

### 4.5 In vivo studies

#### 4.5.1 Experimental animals and study design

The efficacy of wound healing of the Ag/GC‐Ch/PEO/Gel nanofibrous scaffold was evaluated in a BALB/c mice excisional wound splinting model.^59^ All animal experiments performed in this study was according to the ethical guidelines that was approved by the ethical committee of the Shahrekord University of Medical Sciences (IR.SKUMS.REC.1396.78). The experiment was conducted on nine healthy male BALB/c mice with an average weight of 25 ± 2 g. The animals were randomly divided into three groups: negative control (without treatment), treatment with the Ag/GC‐Ch/PEO/Gel nanofibrous scaffold, and treatment with the Ag/GC‐Ch/PEO/Gel nanofibrous scaffold containing MEFs (*N* = 3)

#### 4.5.2 Skin wound creation

The hair of the mice was removed the day before surgery. Diluted cefazolin (0.04 ml) was intraperitoneally (IP) injected into the animals for prophylaxis. General anesthesia was induced with ketamine/xylazine IP injection. Skin surface disinfection was performed using povidone‐iodine, followed by rinsing with 70% (vol/vol) ethanol. Each mouse was placed on its side on a sterile sheet. The dorsal skin of the chest was pulled with two fingers from the midline. Two layers of folded skin were punched with a 5 mm diameter biopsy punch. Two symmetrical full‐thickness excisional wounds were subsequently created on both sides of the midline. An instant‐bonding adhesive was spread on one side of the splinting silicone rings (15 mm external diameter and 5 mm internal diameter) and placed around the wound (glue side down). The splint was sutured to the skin with three 5‐0 Vicryl sutures (Ethicon Inc., Raritan, NJ). The use of a splinting ring enabled a tight approximation of the skin around the wound and prevented local skin contraction. This enabled the wound to heal through the formation of granulation tissue and re‐epithelialization.

The nanofibrous scaffolds to be tested were punched to match the exact size of the ring (5 mm diameter) and placed on the designated wound. For the MEF‐loaded Ag/GC‐Ch/PEO/Gel nanofibrous scaffold, 1 × 10^5^ MEFs were seeded on each scaffold 24 h prior to grafting and kept in an incubator until grafting. Each wound was dressed with Tegaderm transparent dressing (3 M Corp., Maplewood, MN) and a self‐adhering elastic bandage. The mice were placed in separate cages under a warming lamp until they completely recovered from anesthesia. The mice were housed in individual cages with clean facilities to avoid biting wounds. Each mouse was checked daily to ensure that the bandage remained on the wound. On days 3, 7, 14, and 21, each wound bandage was uncovered for measurement of the wound size. Measurement was performed using ImageJ software based on a photograph of the individual wound. The percentage of wound closure was calculated with the equation: ((Original wounds area − Actual wounds area)/(Original wounds area) × 100). Because the original wound size matched the internal diameter of the splinting silicone ring exactly, the size of the splinting ring was considered the original wound size.^59^

#### 4.5.3 Histopathological evaluation

The mice were euthanized 21 days after treatment. The skin tissue from each mouse was harvested, fixed in 10% buffered formalin (pH 7.26) for 48 h, and processed for light microscopy. A microtome was used to cut 5 μm thick sections, which were stained with hematoxylin & eosin (H&E) or Masson's trichrome (MT).^60^, ^61^ Epithelialization, collagen synthesis, and neo‐angiogenesis were observed using an optical light microscope (Olympus BX51; Olympus, Tokyo, Japan) equipped with a digital camera. The images were interpreted by an independent histopathologist.

### 4.6 Statistical analysis

Statistical analyses were performed using a one‐way analysis of variance followed by post hoc Tukey's test. Data analyses were performed using GraphPad Prisma 9 software (San Diego, CA). A *p*‐value below 0.05 was considered statistically significant.

## 5 CONCLUSION

The present work demonstrated that mouse embryonic fibroblasts‐loaded Ag/GC‐Ch/PEO/Gel nanofibrous scaffolds enhanced the cutaneous wound healing process. Nanoscopical crystalline GC and Ag‐doped GC powders were prepared. These bioactive powders were then used to fabricate nanofibrous scaffolds containing Ch/PEO/Gel through electrospinning. In vitro evaluation showed that GC‐Ch/PEO/Gel and Ag/GC‐Ch/PEO/Gel possess antibacterial properties but minimal biofilm eradication capability to potentially relieve the bacterial load on the skin. The nanofibrous scaffolds were biocompatible, hemocompatible, nonimmunogenic, and promoted cell attachment and proliferation. In the in vivo experiment, the mouse embryonic fibroblasts‐loaded Ag/GC‐Ch/PEO/Gel nanofibrous scaffold exhibited adequate wound healing activity with improved angiogenesis, collagen synthesis as well as regeneration of sebaceous glands and hair follicles in a murine full‐thickness excision wound model. The results suggest that nanofibrous scaffolds containing GC or Ag/GC yield acceptable results in skin regeneration and eradication of wound surface infection. The potential of these scaffolds as skin substitutes requires more rigorous in vivo validation using large animal models.

## AUTHOR CONTRIBUTIONS

**Esmaeel Sharifi:** Visualization (equal), Investigation (lead); Methodology (equal); Writing – review and editing (lead). **Seyede Athar Sadati:** Investigation (equal); Methodology (equal). **Satar Yousefiasl:** Investigation (equal); Methodology (equal); Data curation (equal); formal analysis (equal); writing – original draft (equal); writing – review and editing (equal); figures preparation. **Rossella Sartorius:** Methodology (equal); writing – original draft (equal); writing – review and editing (equal). **Mahdi Zafari:** Formal analysis (equal); investigation (equal); methodology (equal). **Leila Rezakhani:** Methodology (equal). **Morteza Alizadeh:** Methodology (equal). **Ehsan Nazarzadeh Zare:** Methodology (equal); writing – review and editing (equal). **Shadi Omidghaemi:** Methodology (equal). **Fatemeh Ghanavatinejad:** Data curation (equal). **Mohammad‐Saeid Jami:** Visualization (equal). **Erfan Salahinejad:** Formal analysis (equal); methodology (equal); writing – review and editing (equal). **Hadi Samadian:** Investigation (equal). **Ana Cláudia Santos:** Methodology (equal); writing – review and editing (equal). **Piergiuseppe De Berardinis:** Investigation (equal). **Abbas Shafiee:** Writing – review and editing (equal). **Franklin R. Tay:** Writing – review and editing (equal). **Samiramis Pourmotabed:** Visualization (equal),  Methodology (equal);  Writing – review and editing (equal). **Pooyan Makvandi:** Methodology (equal), Writing – review and editing (lead).

## CONFLICT OF INTEREST

The authors declare no conflict of interest associated with this work.

### PEER REVIEW

The peer review history for this article is available at https://publons.com/publon/10.1002/btm2.10386.

## Data Availability

The data that support the findings of this study are available from the corresponding author upon reasonable request.
