# Erratum to “Cell loaded hydrogel containing Ag‐doped bioactive glass–ceramic nanoparticles as skin substitute: Antibacterial properties, immune response, and scarless cutaneous wound regeneration”

**DOI:** 10.1002/btm2.10543

**Published:** 2023-07-14

Sharifi, E, Sadati, SA, Yousefiasl, S, Sartorius, R, Zafari, M, Rezakhani, L, Alizadeh, M, Nazarzadeh Zare, E, Omidghaemi, S, Ghanavatinejad, F, Jami, M‐S, Salahinejad, E, Samadian, H, Paiva‐Santos, AC, De Berardinis, P, Shafiee, A, Tay, FR, Pourmotabed, S, and Makvandi, P. (2022). Cell loaded hydrogel containing Ag‐doped bioactive glass–ceramic nanoparticles as skin substitute: antibacterial properties, immune response, and scarless cutaneous wound regeneration. Bioeng Transl Med, 7(3): e10386. doi:10.1002/btm2.10386.

This erratum corrects the mistaken duplication of the sample shown in Figure 5b, which was inadvertently duplicated from 5c. These are different sample sets and Figure 5b is corrected in this erratum.

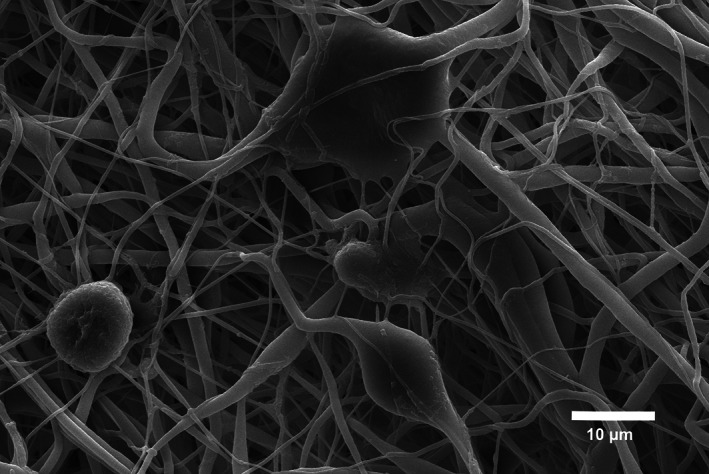

We apologize for this error.

